# Genetic polymorphisms in *MTR* are associated with non-syndromic congenital heart disease from a family-based case-control study in the Chinese population

**DOI:** 10.1038/s41598-019-41641-z

**Published:** 2019-03-25

**Authors:** Changfei Deng, Ying Deng, Liang Xie, Li Yu, Lijun Liu, Hanmin Liu, Li Dai

**Affiliations:** 1 National Center for Birth Defects Monitoring, West China Second University Hospital, Sichuan University, Chengdu, Sichuan China; 2 Key Laboratory of Birth Defects and Related Diseases of Women and Children (Sichuan University), Ministry of Education, Chengdu, Sichuan China; 3 Department of Pediatric Respiration, West China Second University Hospital, Sichuan University, Chengdu, Sichuan China; 4 The Vascular Remodeling and Developmental Defects Research Unit, West China Institute of Women and Children’s Health, West China Second University Hospital, Sichuan University, Chengdu, Sichuan China; 5 Department of Pediatric Cardiology, West China Second University Hospital, Sichuan University, Chengdu, Sichuan China

## Abstract

Genetic polymorphisms of folate pathway genes have been reported to be associated with congenital heart diseases (CHDs); however, the results remain conflicting. We conducted a family-based case-control study, which included160 CHD case-parent triads and 208 control-parent triads to explore the association of 18 genetic variants of seven folate metabolism-related genes with the risk of CHDs. The *MTR* C allele of rs1770449 (OR = 1.961, 95%CI: 1.379–2.788) and the *MTR* A allele of rs1050993 (OR = 1.994, 95%CI: 1.401–2.839) in infants were associated with an increased risk of CHDs. Over-transmission of SNPs rs1770449 and rs1050993 and haplotype CAA (rs1770449-rs1805087-rs1050993) in *MTR* were detected in total CHDs. The above mentioned associations of *MTR* with CHDs were also observed in septal defects and conotruncal heart defects subgroups. Without maternal periconceptional folate intake, the risk of CHDs among women carrying the rs1770449 “CT or CC” genotype or the rs1050993 “AG or AA” genotype in *MTR* was 3.262(95%CI: 1.656–6.429) or 3.263(95%CI: 1.656–6.429) times greater than the aOR in women carrying wild genotype, respectively. Our study suggests that *MTR* polymorphisms (rs1770449 and rs1050993) may be associated with the risk of CHDs and modify the relation between maternal folate intake and CHDs.

## Introduction

Congenital heart diseases (CHDs) represent the most common birth defect and the leading cause of infant deaths worldwide, affecting 0.6–9.1 per 1000 live births with substantial geographic variations^1^. In China, the prevalence of CHD is 8.98 per 1000 live births^2^ and approximately 1 to 4 of every 1000 births with an upward trend during 2000–2011^3^. The aetiology of non-syndromic CHD remains unknown. Many studies over the past decades suggested that the interaction of genetic and environmental factors contributed to the increasing risk of CHDs^4–6^, however, the results are still inconsistent^7^.

It is generally accepted that maternal periconceptional supplementation with folic acid could contribute to a 40–60% reduction in CHDs^8–10^. Furthermore, the results of some association studies on SNPs in the folate metabolism pathway have shown that several genetic variants in *MTHFR, MTR* and *MTRR* might be associated with CHDs^11,12^. However, several other studies reported contrasting results due to differences in study population, design method, etc. These results indicate that more studies are needed to elucidate the mechanism of genetic factors and maternal folic acid intake in CHDs^7,13^.

In the present study, we hypothesized that the perturbations of folate pathway genes are associated with a susceptibility to CHDs. A family-based case-control study was performed to illustrate (1) case-control association, (2) transmission disequilibrium and (3) gene-environment interaction between genetic variants of seven key enzyme genes in the folate metabolic pathway and the risk of CHDs.

## Results

### Demographic characteristics

In our study, a total of 368 complete triads were analysed (160 CHD case-parent triads and 208 control-parent triads). Significant differences were found in case and control groups with respect to maternal age, maternal education level, residence, household income and maternal folate intake (Table 1).

**Table 1:** Characteristics in the CHD case and control groups.

Characteristics	Cases		Controls		*χ* ^2^	*P*
	No.	%	No.	%		
Maternal age(yrs)					21.391	<**0.001**
<25	33	20.63	13	6.25		
25–29	75	46.88	92	44.23		
30–34	41	25.63	79	37.98		
≥35	11	6.88	24	11.54		
Maternal education level					41.334	<**0.001**
Junior school or less	23	14.38	5	2.40		
Senior high school	30	18.75	12	5.77		
College	92	57.50	145	69.71		
Master or advance	15	9.38	46	22.12		
Residence					43.531	<**0.001**
Urban	101	63.13	190	91.35		
Rural	59	36.88	18	8.65		
Household income(rmb monthly)					46.794	<**0.001**
<3000	26	16.25	4	1.92		
3000–5999	41	25.63	24	11.54		
6000–8999	46	28.75	104	50.00		
9000–11999	28	17.50	34	16.35		
≥12000	19	11.88	42	20.19		
Maternal folate intake					8.736	**0.003**
Yes	69	43.13	122	58.65		
No	91	56.88	86	41.35		
Fetal gender					0.004	0.949
Male	81	50.63	106	50.96		
Female	79	49.38	102	49.04		

### Association between infant SNPs and CHDs

None of the genotype frequencies for 18 infant SNPs of seven folate pathway genes in the controls deviated from the Hardy-Weinberg equilibrium(Supplementary Table S1).

Table 2 shows the association between infant SNPs and the risk of CHDs. The C allele of rs1770449 and the A allele of rs1050993 in *MTR* were associated with an increased risk of CHDs(aOR = 1.96, 95%CI: 1.38–2.79; aOR = 1.99, 95%CI: 1.40–2.84) after the false discovery rate (FDR) correction. Under additive model, the SNP rs1770449 and rs1050993 (aOR = 1.87, 95%CI: 1.27–2.76; aOR = 1.90, 95%CI: 1.28–2.80) were associated with an increased risk of CHDs under FDR correction for multiple testing.

**Table 2:** Association between infant MTR genetic polymorphisms and CHDs assuming various genetic models.

Marker	Polymorphisms	Cases		Controls		aOR (95%CI)*	*P* ^$^
		No.	%	No.	%		
rs1770449							
	T	228	71.3	345	82.9	Ref.	
	C	92	28.8	71	17.1	1.961 (1.379, 2.788)	**0.003**
Dominant	TT	82	51.3	141	67.8	Ref.	
	TC or CC	78	48.8	67	32.2	1.835 (1.159, 2.907)	0.130
Recessive	TC or TT	146	91.3	204	98.1	Ref.	
	CC	14	8.8	4	1.9	4.759 (1.452, 15.600)	0.190
Additive	—	—	—	—	—	1.870 (1.267, 2.762)	**0.031**
rs1050993							
	G	228	71.3	346	83.2	Ref.	
	A	92	28.8	70	16.8	1.994 (1.401, 2.839)	**0.003**
Dominant	GG	82	51.3	142	68.3	Ref.	
	GA or AA	78	48.8	66	31.7	1.873 (1.182, 2.969)	0.130
Recessive	GA or GG	146	91.3	204	98.1	Ref.	
	AA	14	8.8	4	1.9	4.759 (1.452, 15.600)	0.190
Additive	—	—	—	—	—	1.898 (1.284, 2.804)	**0.031**

*Odds ratios adjusted by maternal age, maternal education level, fetal gender.

^$^Three models adjusted by multiple testing correction of false discovery rate. 18 polymorphisms in seven genes were covered by this correction.

### Transmission disequilibrium

Table 3 and Supplementary Table S2 present the transmission of genetic variants from heterozygous CHD case-parent triads. Over-transmission was observed in the SNPs rs1770449 and rs1050993 of *MTR* after the adjustment by the permutation test as follows: total CHDs (rs1770449: OR = 2.28,95%CI: 1.51–3.46; rs1050993: OR = 2.28, 95%CI: 1.51–3.46), septal defects (SPD) (rs1770449: OR = 2.32, 95%CI: 1.45–3.71; rs1050993: OR = 2.32, 95%CI: 1.45–3.71), conotruncal heart defects (CTD) (rs1770449:OR = 4.00, 95%CI: 2.07–7.75; rs1050993: OR = 4.00, 95%CI: 2.07–7.75).

**Table 3:** Transmission of MTR variant alleles from heterozygous case-parent triads.

Marker	Subtype	Transmitted	Not transmitted	OR(95%CI)	*χ* ^*2*^	*P*_*m*_^#^
rs1770449	Total	73	32	2.281 (1.506, 3.457)	16.010	**0.001**
	SPD	58	25	2.320 (1.452, 3.708)	13.120	**0.007**
	CTD	44	11	4.000 (2.066, 7.745)	19.800	** <0.001**
rs1050993	Total	73	32	2.281 (1.506, 3.457)	16.010	**0.001**
	SPD	58	25	2.320 (1.452, 3.708)	13.120	**0.007**
	CTD	44	11	4.000 (2.066, 7.745)	19.800	** <0.001**

^#^*P*_m_: *P* value adjusted by permutation test.

Enhanced risk was found in haplotype CAA (rs1770449-rs1805087-rs1050993), while decreased risk was found in haplotype TAG in the total CHD, SPD and CTD groups (Table 4). Transmission of haplotypes in other related genes is shown in Supplementary Table S3.

**Table 4:** Transmission of MTR haplotype from heterozygous case-parent triads.

Haplotype	Subtype	Transmitted	Not transmitted	*χ* ^*2*^	*P*
MTR 3-SNP: rs1770449-rs1805087-rs1050993					
CAA	Total	73	32	16.010	** <0.001**
	SPD	58	25	13.120	** <0.001**
	CTD	44	11	19.800	** <0.001**
TGG	Total	24	31	0.891	0.345
	SPD	17	23	0.900	0.343
	CTD	12	15	0.333	0.564
TAG	Total	53	87	8.257	**0.004**
	SPD	40	67	6.813	**0.009**
	CTD	23	53	11.840	**0.001**

### Interaction between infant SNPs and maternal folate intake

Assuming a dominant genetic model, multiplicative-scale interactions between maternal folate intake and the SNPs rs1770449 (B = −1.152, Wals = 5.743) and rs1050993 (B = −1.116,Wals = 5.386) in *MTR* and the risk of CHDs were observed after adjusting for potential confounders. Without periconceptional maternal folate intake, the risk of CHDs among the women carrying rs1770449 “CT or CC” genotype or rs1050993 “AG or AA” genotype in *MTR* was 3.262(95%CI: 1.656–6.429) or 3.263(95%CI: 1.656–6.429) times greater than the aOR in women carrying wild genotype, respectively.

Further analysis found additive-scale interactions between the above mentioned SNPs (rs1770449: S = 0.066, RERI = −2.227; rs1050993: S = 0.078, RERI = −2.186) and maternal folate intake (Table 5).

**Table 5:** Interaction between maternal folic acid supplement and infant genotypes of MTR gene on the risk of CHDs.

Marker	Genotype	Maternal folate intake	Case	Control	aOR^a^ (95%CI)	*P*_1_^b^	*P*_2_^c^
rs1770449	TT	no	40	60	Ref.	**0.015**	**0.014**
	TT	yes	42	81	1.123 (0.619, 2.039)		
	CT or CC	no	51	26	3.262 (1.656, 6.429)		
	CT or CC	yes	27	41	1.158 (0.585, 2.295)		
rs1050993	GG	no	40	60	Ref.	**0.019**	**0.016**
	GG	yes	42	82	1.109 (0.611, 2.012)		
	AG or AA	no	51	26	3.263 (1.656, 6.429)		
	AG or AA	yes	27	40	1.185 (0.597, 2.354)		

^a^Odds ratios adjusted for maternal age, maternal education level, fetal gender.

^b^P_1_: counted by logistic regression. ^c^P_2_: counted by cross analysis.

## Discussion

In this family-based association study, over-transmission of SNPs rs1770449 and rs1050993 and haplotype CAA (rs1770449-rs1805087-rs1050993) in *MTR* were detected in total CHDs, SPD and CTD groups. Significant interactions between maternal periconceptional folic acid supplementation and foetal SNPs rs1770449 and rs1050993 on the risk of CHDs were observed.

Our results indicated that *MTR* may be associated with the risk of CHDs, which was identified in both case-control and TDT analyses. To our knowledge, these SNPs have not been previously reported in association with CHDs. In a study with 2340 CHD cases and 2270 controls performed in China, researchers found that two regulatory variants of *MTR*, −186T > G and + 905 G > A, were associated with an increased risk of CHD. Both of the minor alleles were correlated with elevated plasma homocysteine concentrations, indicating a genetic component for hyperhomocysteinaemia^11^. Previous studies indicated that CHD is related to a high homocysteine (Hcy) level^14^ or hyperhomocysteinaemia, which leads to a cardiovascular damage in the early development period^15,16^. *MTR* is a key enzyme in folate metabolism that catalyses the transfer of a methyl group from 5-methyltetrahydrofolate to Hcy and removes Hcy to methionine^7^. A study in the Thai population found an increased risk of breast cancer for homozygotes in the *MTR* SNPs (rs1770449 and rs1050993) with the OR = 2.21 and OR = 2.24, respectively^17^. Moreover, homozygous *MTR* knock-out mice are embryonically lethal, suggesting that *MTR* activity is essential for early embryonic development^18^.

In addition, the activation of *MTR* requires for *MTRR* gene and it has been demonstrated that the *MTRR* c.56 + 781 A > C variant results in functionally reduced *MTRR* expression at the transcriptional level^12^. Furthermore, *MTRR* regenerates the functional status of *MTR* via the chemical reduction of vitamin B12^19^. Therefore, both *CUBN*, which encodes the intrinsic factor-vitamin B12 receptor, and *TCNI*, a vitamin B12 binding protein, participate in the remethylation of Hcy^20,21^. In this study, our finding did not support a role of *MTRR*, *CUBN* and other included folate pathway genes in modifying the risk of CHDs in Chinese women, implying that studies with larger sample sizes are needed.

In our research, we further analysed the interaction of folate metabolism pathway genes and maternal folate intake on the risk of CHD and found that two SNPs (rs1770449 and rs1050993) in infants can modify the association between maternal folic acid intake and CHD risk. Folate is a kind of B vitamin and one-carbon donor, that plays an important role in DNA synthesis and cell division^22^. Nonetheless, folate cannot be synthesized in the body. Our study indicated that periconceptional folic acid supplementation was associated with a decreased risk of CHD, which is consistent with other studies^23–25^. Furthermore, our results suggested that this association could be modified by variants of the infant *MTR* gene. Hobbs reported that obese women carrying the TT genotype of *MTHFR* were 4.6 times more likely to have an affected pregnancy compared with normal-weight women carrying a CC genotype^4^. Women who smoked, were in the highest quartile of homocysteine concentration and had the MTHFR CC genotype, had a 12-fold increased CHD risk compared with the reference group^26^. Other studies found an interaction between RFC1 80GG and folic acid intake on the risk of neural tube defects^27^.

There are some major strengths in this study. First, we performed a combination of case-control and case-parental control design, which allows both case-control association and TDT analysis of the cases. TDT, a generally recommended method, can effectively avoid the bias caused by population stratification^28,29^. The combination of the two study designs is cost-effective and robust, especially when the case-control and TDT results coincide with each other, offering greater confidence in the result^30^. Second, both multivariate logistic regression analysis and cross analysis were used to analyze the interaction between maternal folate intake and SNPs on the risk of CHDs. Researchers argued that interaction estimated as departure from additivity could better reflect a biologic interaction^31,32^. Third, the epidemiological data were collected in the first trimester to avoid recall bias. Fourth, the CHD cases were validated by following up one year after delivery. The limitations include that the study was performed in a single hospital, and the data about maternal environmental exposure were collected by self-reporting, not by the biomarker-based approach.

In summary, our study indicated that *MTR* SNPs rs1770449 and rs1050993 and their haplotypes may be associated with the risk of CHDs. To the best of our knowledge, no other study has reported the association between these SNPs and the risk of CHDs. Moreover, rs1770449 and rs1050993 could also modify the association between maternal folic acid intake and CHD risk, which indicated that mothers with foetuses carrying the C allele of rs1770449 and the A allele rs1050993 should take folic acid per their doctor’s recommendation. Our findings provide new evidence about CHD pathogenesis and enrich the knowledge of the impact of folate pathway genetic variants on CHDs, which are of great value for CHD clinical intervention. The results need to be verified in further large-scale studies and functional studies.

## Materials and Methods

### Study population and data collection

The study was performed at the West China Second University Hospital from January 2014 to September 2015. A total of 160 CHD case-parent triads and 208 control-parent triads were recruited. Mothers with a singleton pregnancy diagnosed with CHD by echocardiography without extracardiac abnormalities, syndromic diseases and chromosomal aberrations were selected in the case group. Stillbirths were aborted according to standard process, and livebirths were followed up one year after delivery. Foetuses or infants with cardiomyopathy, cardiac rhabdomyoma, single umbilical artery or arrhythmia were excluded from the study. Mothers with a singleton pregnancy without any congenital malformations were recruited to the control group and matched by residence area and ethnicity.

All cases were classified into three subgroups based on the origin of heart development as follows: (1) septal defects (SPD); (2) conotruncal heart defects (CTD), including conotruncal, right-sided obstructive, left-sided obstructive heart defects and (3) other cardiac abnormalities. When performing subgroup analyses, we focused on SPD and CTD groups, because the sample size of other cardiac abnormalities was too small to count.

All the participants accepted a face-to-face interview in the first, second and third trimesters and 30 days after delivery. The questionnaires were composed of parental epidemiological characteristics, maternal diet and nutrition, living and working environment, etc. Maternal folate intake means mothers taking folic acid over 90 days or more during the 3 months before pregnancy to the first trimester^33^.

This research was approved by the Ethics Committee of West China Second University Hospital and was based on the tenets of the Declaration of Helsinki. Informed consent was collected from all participants.

### Marker selection and genotyping

Eighteen SNPs in seven folate pathway genes, including *MTR, MTRR, BHMT, BHMT*2*, CUBN,TCN1*, and *TCN*2 were selected based on (1) minor allele frequency(>0.05) and (2) association with CHDs or other congenital abnormalities in past studies^7,11,21,34^. Genomic DNA was extracted from peripheral blood according to the manufacturer (QIAamp DNA Blood Mini Kit), and SNPs were genotyped using a commercial custom-by-design 2 × 48-Plex SNPscan^TM^ Kit (Cat#: G0104, Genesky Biotechnologies Inc., Shanghai, China), as described previously^35^. Genotyping was performed in a single-blind approach and the sample grouping was unknown to laboratory staff. Ten percent of samples were randomly repeated for genotyping.

### Statistical analysis

The demographic characteristics of the different groups were compared by chi-squared test using Statistical Package for Social Sciences (SPSS) version 21.0 software (SPSS Inc., IBM, Chicago, USA). A chi-squared test was performed to investigate the associations between allele frequencies and CHDs, and unconditional logistic regression analysis was used to investigate the associations between genotypes and CHDs assuming various genetic models (dominant, recessive and additive), respectively, in case and control groups using Plink software (http://pngu.mgh.harvard.edu/~purcell/plink/) in accordance with the Hardy–Weinberg equilibrium in the controls. False discovery rate (FDR) correction of multiple hypothesis testing was performed^36^.

Interactions between maternal folate supplement and infant genotypes on the risk of CHDs were assessed by multiple logistic regression analysis and cross analysis and adjusted by maternal age, maternal education level and foetal gender. Regression coefficient (B), value of Wald test (Wals), synergy index (S) and relative excess risk of interaction (RERI) were counted.

Transmission disequilibrium test(TDT) of alleles and haplotypes in 160 complete case-parent triads were analysed by Plink software. TDT was adjusted by permutation test and 10,000 permutations were conducted. The linkage disequilibrium (LD) patterns and haplotype structures were estimated using Haploview 4.2 software^37^. Two-sided *P* < 0.05 was considered statistically significant.

## Supplementary information

supplementary information
