# Supplementary material for: Genetic polymorphisms in MTR are associated with non-syndromic congenital heart disease from a family-based case-control study in the Chinese population
Source: Sci Rep. 2019 Mar 25;9:5065. doi: 10.1038/s41598-019-41641-z (PMC6433945; doi:10.1038/s41598-019-41641-z)
Supplement: Supplementary file 1 — supplementary information [file 41598_2019_41641_MOESM1_ESM.pdf]

Genetic polymorphisms in *MTR* are associated with non-syndromic congenital heart disease from a family-based case-control study in the Chinese population

Changfei Deng[1,2], Ying Deng[1,2], Liang Xie[2,3,4], Li Yu[5], Lijun Liu[2], Hanmin Liu[2,3,4], Li Dai[1,2*]

[1] National Center for Birth Defects Monitoring, West China Second University Hospital, Sichuan University, Chengdu, Sichuan, China

[2] Key Laboratory of Birth Defects and Related Diseases of Women and Children （Sichuan University）, Ministry of Education , Chengdu, Sichuan, China

[3] Department of Pediatric Respiration, West China Second University Hospital, Sichuan University, Chengdu, Sichuan, China

[4] The Vascular Remodeling and Developmental Defects Research Unit, West China Institute of Women and Children's Health, West China Second University Hospital, Sichuan University, Chengdu, Sichuan, China

[5] Department of Pediatric Cardiology, West China Second University Hospital, Sichuan University, Chengdu, Sichuan, China

---

Changfei Deng and Ying Deng contributed equally to this work.

*Correspondence and requests for materials should be addressed to Li Dai (email: daili@scu.edu.cn)

Supplementary Table S1 SNP locations, alleles, and frequencies

Gene	Marker	allele	Chr.	Chr. Position	Location	Functional Change	MAF[d]	HWP[e]
MTR	rs1770449	T/C	1	237038161	intron24	/	0.232	0.461
MTR	rs1805087	G/A	1	237048500	nonsynon_exon26	p.Asp919Gly	0.218	1.000
MTR	rs1050993	G/A	1	237062305	3'-UTR	/	0.236	0.461
MTRR	rs326119	C/A	5	7870083	intron1	/	0.368	0.884
MTRR	rs1801394	G/A	5	7870973	nonsynon_exon2	p.Ile22Met	0.364	0.143
MTRR	rs1532268	T/C	5	7878179	nonsynon_exon5	p.Ser175Leu	0.270	0.384
MTRR	rs162036	G/A	5	7885959	nonsynon_exon7	p.Lys350Arg	0.253	0.668
MTRR	rs162049	G/A	5	7893121	intron11	/	0.258	0.404
MTRR	rs10380	T/C	5	7897191	nonsynon_exon14	p.His595Tyr	0.219	0.634
BHMT2	rs682985	T/C	5	78373431	synon_exon2	p.=(Asp54Asp)	0.488	0.664
BHMT2	rs625879	C/A	5	78381689	intron7	/	0.489	0.770
BHMT	rs651852	T/C	5	78409060	intron1	/	0.412	0.660
BHMT	rs3733890	G/A	5	78421959	nonsynon_exon6	p.Arg239Gln	0.219	0.532
CUBN	rs11254363	G/A	10	17130693	intron14	/	0.230	1.000
CUBN	rs1801222	G/A	10	17156151	nonsynon_exon8	p.Phe253Ser	0.201	0.296
TCN1	rs526934	G/A	11	59633493	intron1	/	0.198	0.269
TCN2	rs9606756	G/A	22	31006860	nonsynon_exon2	p.Ile23Val	0.113	1.000
TCN2	rs1801198	G/C	22	31011610	nonsynon_exon6	p.Arg259Pro	0.420	1.000

d: Minor allele frequence. e: *P* value of Hardy-Weinberg equilibrium.

Supplementary Table S2 Transmission of variant alleles from heterozygous case-parent triads

Gene	Marker	Subtype	Transmitted	Not transmitted	OR(95%CI)	χ^2	P_m[#]
MTR	rs1805087	Total	24	31	0.774(0.454,1.319)	0.891	0.345
		SPD	17	23	0.739(0.395,1.383)	0.900	0.343
		CTD	12	15	0.800(0.375,1.709)	0.333	0.564
MTRR	rs326119	Total	72	62	1.161(0.827,1.631)	0.746	1.000
		SPD	55	48	1.146(0.778,1.688)	0.476	1.000
		CTD	43	30	1.433(0.899,2.285)	2.315	0.963
MTRR	rs1801394	Total	55	55	1.000(0.688,1.453)	0.000	1.000
		SPD	39	43	0.907(0.588,1.399)	0.195	1.000
		CTD	30	28	1.071(0.640,1.793)	0.069	1.000
MTRR	rs1532268	Total	47	48	0.979(0.655,1.464)	0.011	1.000
		SPD	31	41	0.756(0.474,1.206)	1.389	1.000
		CTD	33	24	1.375(0.813,2.326)	1.421	0.999
MTRR	rs162036	Total	47	54	0.870(0.589,1.287)	0.485	1.000
		SPD	36	37	0.973(0.615,1.539)	0.014	1.000
		CTD	17	31	0.548(0.304,0.991)	4.083	0.686
MTRR	rs162049	Total	80	73	1.096(0.798,1.505)	0.320	1.000
		SPD	65	55	1.182(0.825,1.692)	0.833	1.000
		CTD	45	37	1.216(0.787,1.879)	0.781	1.000
MTRR	rs10380	Total	43	49	0.878(0.583,1.322)	0.391	1.000
		SPD	33	35	0.943(0.586,1.517)	0.059	1.000
		CTD	16	30	0.533(0.291,0.978)	4.261	0.644
BHMT2	rs682985	Total	67	81	0.827(0.598,1.143)	1.324	1.000
		SPD	54	64	0.844(0.587,1.212)	0.848	1.000
		CTD	29	43	0.674(0.421,1.080)	2.722	0.922
BHMT2	rs625879	Total	69	81	0.852(0.618,1.174)	0.960	1.000
		SPD	55	63	0.873(0.608,1.253)	0.542	1.000
		CTD	31	43	0.721(0.454,1.144)	1.946	0.996
BHMT	rs651852	Total	70	82	0.854(0.621,1.174)	0.947	1.000
		SPD	55	64	0.859(0.599,1.232)	0.681	1.000
		CTD	32	44	0.727(0.461,1.147)	1.895	0.997
BHMT	rs3733890	Total	66	68	0.971(0.692,1.362)	0.030	1.000
		SPD	48	50	0.960(0.646,1.427)	0.041	1.000
		CTD	34	34	1.000(0.622,1.609)	0.000	1.000
CUBN	rs11254363	Total	9	11	0.818(0.339,1.974)	0.200	1.000
		SPD	8	11	0.727(0.293,1.808)	0.474	1.000
		CTD	5	3	1.667(0.398,6.974)	0.500	1.000
CUBN	rs1801222	Total	41	52	0.789(0.524,1.187)	1.301	1.000
		SPD	29	39	0.744(0.460,1.202)	1.471	0.999
		CTD	22	31	0.710(0.411,1.226)	1.528	0.999
TCN1	rs526934	Total	63	45	1.400(0.955,2.052)	3.000	0.931
		SPD	53	37	1.432(0.941,2.180)	2.844	0.947
		CTD	30	26	1.154(0.683,1.951)	0.286	1.000
TCN2	rs9606756	Total	4	4	1.000(0.250,3.998)	0.000	1.000
		SPD	4	3	1.333(0.298,5.957)	0.143	1.000
		CTD	2	0	NA	2.000	0.996
TCN2	rs1801198	Total	70	92	0.761(0.558,1.038)	2.988	0.934
		SPD	50	70	0.714(0.497,1.027)	3.333	0.861
		CTD	33	52	0.635(0.410,0.982)	4.247	0.652

[#] P_m： P value adjusted by permutation test.

Supplementary Table S3 Transmission of haplotype from heterozygous case-parent triads

Haplotype	Subtype	Transmitted	Not transmitted	χ^2	*P*
MTRR	2-SNP: rs326119-rs1801394				
AG	Total	56	55	0.009	0.924
	SPD	40	43	0.110	0.740
	CTD	30	28	0.069	0.793
CA	Total	72	61	0.910	0.340
	SPD	55	47	0.625	0.429
	CTD	43	30	2.315	0.128
AA	Total	62	73	0.896	0.344
	SPD	48	52	0.158	0.691
	CTD	26	41	3.358	0.067
MTRR	2-SNP: rs1532268-rs162036				
CG	Total	47	54	0.485	0.486
	SPD	36	37	0.014	0.907
TA	Total	47	48	0.011	0.918
	SPD	31	41	1.389	0.239
CA	Total	80	72	0.421	0.516
	SPD	65	54	1.017	0.313
MTRR	2-SNP: rs162049-rs10380				
AT	Total	43	49	0.391	0.532
	SPD	33	35	0.059	0.808
	CTD	16	30	4.261	0.039
GC	Total	80	73	0.320	0.572
	SPD	65	55	0.833	0.361
	CTD	45	37	0.781	0.377
AC	Total	69	70	0.007	0.932
	SPD	47	55	0.628	0.428
	CTD	41	35	0.474	0.491
BHMT2	2-SNP: rs682985-rs625879				
CA	Total	67	81	1.324	0.250
	SPD	54	64	0.848	0.357
	CTD	29	43	2.722	0.099
TC	Total	81	69	0.960	0.327
	SPD	63	55	0.542	0.462
	CTD	43	31	1.946	0.163
